# Frequency and impact on renal transplant outcomes of urinary tract infections due to extended-spectrum beta-lactamase-producing *Escherichia coli* and *Klebsiella species*

**DOI:** 10.3389/fmed.2024.1329778

**Published:** 2024-02-15

**Authors:** Jakob E. Brune, Michael Dickenmann, Daniel Sidler, Laura N. Walti, Déla Golshayan, Oriol Manuel, Fadi Haidar, Dionysios Neofytos, Aurelia Schnyder, Katia Boggian, Thomas F. Mueller, Thomas Schachtner, Nina Khanna, Stefan Schaub, Caroline Wehmeier, the Swiss Transplant Cohort Study, Patrizia Amico, John-David Aubert, Adrian Bachofner, Vanessa Banz, Sonja Beckmann, Guido Beldi, Christoph Berger, Ekaterine Berishvili, Annalisa Berzigotti, Pierre-Yves Bochud, Sanda Branca, Heiner Bucher, Anne Cairoli, Emmanuelle Catana, Yves Chalandon, Sabina De Geest, Sophie De Seigneux, Michael Dickenmann, Joëlle Lynn Dreifuss, Michel Duchosal, Thomas Fehr, Sylvie Ferrari-Lacraz, Jaromil Frossard, Christian Garzoni, Déla Golshayan, Nicolas Goossens, Fadi Haidar, Jörg Halter, Dominik Heim, Christoph Hess, Sven Hillinger, Hans Hirsch, Patricia Hirt, Linard Hoessly, Günther Hofbauer, Uyen Huynh-Do, Franz Immer, Michael Koller, Andreas Kremer, Christian Kuhn, Bettina Laesser, Frédéric Lamoth, Roger Lehmann, Alexander Leichtle, Oriol Manuel, Hans-Peter Marti, Michele Martinelli, Valérie McLin, Katell Mellac, Aurélia Merçay, Karin Mettler, Nicolas Müller, Ulrike Müller-Arndt, Beat Müllhaupt, Mirjam Nägeli, Graziano Oldani, Manuel Pascual, Jakob Passweg, Rosemarie Pazeller, Klara Posfay-Barbe, David Reineke, Juliane Rick, Anne Rosselet, Simona Rossi, Rössler, Silvia Rothlin, Frank Ruschitzka, Thomas Schachtner, Stefan Schaub, Alexandra Scherrer, Dominik Schneidawind, Aurelia Schnyder, Macé Schuurmans, Simon Schwab, Thierry Sengstag, Federico Simonetta, Jürg Steiger, Guido Stirniman, Ueli Stürzinger, Christian Van Delden, Jean-Pierre Venetz, Jean Villard, Julien Vionnet, Madeleine Wick, Markus Wilhlem, Patrick Yerly

**Affiliations:** ^1^Clinic for Transplantation Immunology and Nephrology, University Hospital Basel, Basel, Switzerland; ^2^Clinic for Nephrology, Bern University Hospital, Bern, Switzerland; ^3^Department of Infectious Diseases, Inselspital, Bern University Hospital, Bern, Switzerland; ^4^Transplantation Center, Lausanne University Hospital, Lausanne, Switzerland; ^5^Infectious Diseases Service, Lausanne University Hospital, Lausanne, Switzerland; ^6^Nephrology and Hypertension Service, Division of Medicine, University Hospital Geneva, Geneva, Switzerland; ^7^Transplant Infectious Disease Service, Division of Infectious Diseases, University Hospital Geneva, Geneva, Switzerland; ^8^Clinic for Nephrology, Kantonsspital St. Gallen, St. Gallen, Switzerland; ^9^Division of Infectious Diseases and Hospital Epidemiology, Kantonsspital St. Gallen, St. Gallen, Switzerland; ^10^Clinic for Nephrology, University Hospital Zürich, Zürich, Switzerland; ^11^Division of Infectious Diseases and Hospital Epidemiology, University Hospital Basel, Basel, Switzerland; ^12^Transplantation Immunology, Department of Biomedicine, University of Basel, Basel, Switzerland

**Keywords:** kidney transplantation, urinary tract infection, Enterobacterales, *E. coli*, Klebsiella, ESBL − extended-spectrum beta-lactamase, graft survival

## Abstract

**Background:**

*Enterobacterales* are often responsible for urinary tract infection (UTI) in kidney transplant recipients. Among these, *Escherichia coli* or *Klebsiella species* producing extended-spectrum beta-lactamase (ESBL) are emerging. However, there are only scarce data on frequency and impact of ESBL-UTI on transplant outcomes.

**Methods:**

We investigated frequency and impact of first-year UTI events with ESBL *Escherichia coli* and/or *Klebsiella species* in a prospective multicenter cohort consisting of 1,482 kidney transplants performed between 2012 and 2017, focusing only on 389 kidney transplants having at least one UTI with *Escherichia coli* and/or *Klebsiella species*. The cohort had a median follow-up of four years.

**Results:**

In total, 139/825 (17%) first-year UTI events in 69/389 (18%) transplant recipients were caused by ESBL-producing strains. Both UTI phenotypes and proportion among all UTI events over time were not different compared with UTI caused by non-ESBL-producing strains. However, hospitalizations in UTI with ESBL-producing strains were more often observed (39% versus 26%, *p* = 0.04). Transplant recipients with first-year UTI events with an ESBL-producing strain had more frequently recurrent UTI (33% versus 18%, *p* = 0.02) but there was no significant difference in one-year kidney function as well as longer-term graft and patient survival between patients with and without ESBL-UTI.

**Conclusion:**

First-year UTI events with ESBL-producing *Escherichia coli* and/or *Klebsiella species* are associated with a higher need for hospitalization but do neither impact allograft function nor allograft and patient survival.

## 1 Introduction

Infections are still an important cause of morbidity and mortality following kidney transplantation (1–3). Since the most intense immunosuppression is applied during the first year post-transplant, the incidence of infections is highest during this period (4). Urinary tract infection (UTI) comprises the most frequently observed type of infection (5, 6). As causative pathogens, *Enterobacterales* play a major role and are responsible for UTI in 50 to 80% of cases, mostly caused by *Escherichia (E.) coli* and *Klebsiella* *spp*. (7, 8). While susceptible strains can be treated by commonly available antibiotics, the increasing percentage of infections by *Enterobacterales* producing extended-spectrum beta-lactamase (ESBL) often requires treatment with carbapenems that need to be applied intravenously and are more expensive compared with most standard antibiotics. In addition, infections with ESBL-producing strains have been associated with a higher clinical and economic burden of disease, longer duration of hospitalization as well as increased mortality (9, 10).

Kidney transplant recipients might be particularly prone to develop UTI with ESBL-producing *Enterobacterales* due to the antimicrobial escape pressure provoked by the use of antibiotic prophylaxis as well as early empiric treatment in case of suspected infection. In addition, UTI with ESBL-producing strains may affect the outcome of transplantation. Only few single-center studies have so far investigated UTI by ESBL-producing *Enterobacterales* in kidney transplant recipients in more detail. In a cohort of kidney transplant recipients from Paris (France), Pilmis et al. described an 11% prevalence of bacteriuria with ESBL-producing strains and about 50% of patients developed an UTI (11). In another larger study from Spain, Bodro et al. found a higher proportion of UTI caused by ESBL-producing *Enterobacterales* among transplant recipients with recurrent episodes of UTI versus non-recurrent UTI (12). Brakemeier et al. reported a lower patient survival but similar death-censored allograft survival in patients with ESBL-UTI compared with a control group (13). Notably, patients of all cohorts were transplanted about or even more than 10 years ago and were, as expected, frequently on cyclosporine and mTOR inhibitors for maintenance immunosuppression, which does not represent the current standard of immunosuppression. Only the study of Brakemeier et al. did investigate the role of UTI with ESBL-producing *Enterobacterales* with respect to graft and patient survival. Therefore, the aim of this study was to describe the frequency as well as the impact of first-year ESBL-UTI on transplant outcomes in a large nationwide contemporary cohort of kidney transplant recipients.

## 2 Materials and methods

### 2.1 Data source

The Swiss Transplant Cohort Study (STCS) is a multicenter, observational and long-term follow-up cohort project recruiting solid organ transplant recipients at all six Swiss transplant centers since 2008. Design, methodology and details on the cohort of the STCS have been previously published (14, 15). This study (project number FUP168) was nested within the STCS and separately approved by the ethics committee of Northwestern and Central Switzerland (www.eknz.ch; project ID 2021-00360). Detailed patient- and transplant-specific data, including infectious disease episodes, are prospectively collected in the STCS. In addition, information on ESBL-production of the causative pathogen has been captured since 2012.

### 2.2 Study cohort

**Figure 1:**
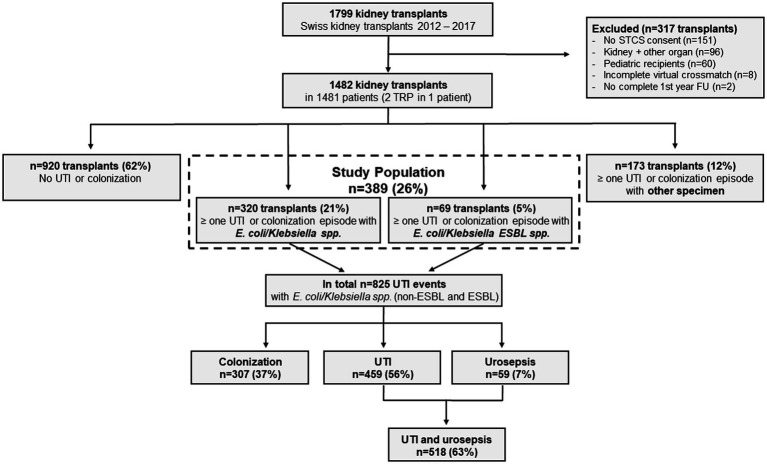
Study flowchart. ESBL, extended-spectrum beta-lactamase; *E. coli, Escherichia coli*; FU, follow-up; STCS, Swiss Transplant Cohort Study; TRP, transplantation; UTI, urinary tract infection.

Between January 2012 and December 2017, 1799 kidney transplantations were performed in Switzerland. For this study, 317 (18%) transplantations were excluded for the following reasons: no STCS consent (*n* = 151), multiorgan transplants (*n* = 96), pediatric recipients (*n* = 60), missing pre-transplant donor-specific HLA antibody assignment (*n* = 8), no complete first-year follow-up (*n* = 2). This resulted in a cohort consisting of 1,482 transplants in adults eligible for study inclusion (Figure 1). In order to be able to comparatively analyze the impact of first-year ESBL-UTI, we subsequently focused only on transplants experiencing at least one first-year UTI event with *E. coli* and/or *Klebsiella* *spp*. This decision was made because 96% of all infections caused by ESBL-producing strains belonged to these two bacterial species. The final study population consisted of 389 kidney transplants (Figure 1). The median age of the cohort was 56 years (47–64 years) and 58% were women.

### 2.3 Definitions

For this study, the same classification of UTI events as in a previous study of our group was used (7). Briefly, all UTI events were classified by an infectious disease specialist and/or nephrologist based on microbiological cultures, urine analyses, and recorded clinical symptoms as follows:

- Urinary colonization (equivalent to ‘asymptomatic bacteriuria/UTI’) was defined as the presence of bacteria in the urine with ≥10^5^ colony forming units (CFU)/ml in the absence of local and systemic signs or symptoms of infection.
- UTI was defined as the presence of bacteria in the urine with ≥10^5^ CFU/mL in the presence of local and/or systemic signs or symptoms of infection. No distinction between lower UTI (i.e., cystitis) and upper UTI (i.e., pyelonephritis) was recorded in the STCS database.
- Urosepsis was defined as the detection of the same pathogen in urine and blood cultures in the presence of local and/or systemic symptoms of infection.

Recurrent UTI were defined as ≥ three UTI events within the first year. At all six transplant centers, urine cultures were taken in case of leucocyturia and/or symptoms referring to an UTI. Additionally, at one center, urine cultures were taken at each consultation during the first 6 months after transplantation.

### 2.4 Treatment of UTI

At all transplant centers, UTI were consistently treated. Colonizations were only treated in 2/6 centers early after transplantation (for the first 6 months after transplantation and as long as the double J-stent was *in situ*, respectively). At all centers, patients with recurrent UTI underwent thorough clinical work-up for underlying gynecological or urogenital pathologies.

### 2.5 Catheter policy and infection prophylaxis

At all six kidney transplant centers, the allograft recipients received a Foley catheter after transplantation, which was removed between postoperative days 4 and 7. A double J-stent was inserted during transplantation as a standard procedure in 5/6 transplant centers, which was removed between two and eight weeks after transplantation. At all centers, patients received trimethoprim-sulfamethoxazole as pneumocystis prophylaxis for 6 months after transplantation. Additionally, at one transplant center, the patients received antibiotic prophylaxis with either amoxicillin/clavulanic acid or ciprofloxacin until the double J-stent was removed.

### 2.6 Diagnosis of rejection

Transplant biopsies were performed at any time in case of suspected rejection or unexplained graft dysfunction. Only one of the six Swiss transplant centers performed protocol biopsies at month 3 and month 6 on a regular basis. Biopsy-proven rejection episodes were graded according to the Banff 2017 classification, excluding the ‘borderline changes’ category.

### 2.7 Outcomes

Data of the study population were analyzed on the patient level as well as on the UTI level. On the UTI level, we investigated the incidence of infections with *E. coli and/or Klebsiella* *spp*., the proportion of UTI with ESBL-producing strains as well as the frequency of treatment and the risk for hospitalization due to UTI. On the patient level, the investigated outcomes were graft function (i.e., estimated glomerular filtration rate [eGFR] according to the Chronic Kidney Disease Epidemiology Collaboration equation) at one-year post-transplant, occurrence of rejection as well as short- and long-term death-censored allograft and patient survival.

### 2.8 Statistical analysis

JMP Pro version 16 software (SAS Institute Inc., Cary, NC, United States) was used for statistical analysis. Data were visualized by GraphPad Prism version 10 (GraphPad Software, San Diego, CA, United States). Categorical data are presented as counts and/or percentages and were analyzed by chi-square test or Fisher’s exact test as appropriate. Continuous data are shown as median and interquartile ranges (IQR) and compared by Wilcoxon rank sum tests. For all tests, a (two-tailed) value of *p* <0.05 was considered to indicate statistical significance. Survival curves were generated by the Kaplan–Meier method, and the groups compared using the log-rank test.

## 3 Results

### 3.1 Baseline characteristics of patient groups

On the patient level, 389 kidney transplant recipients experienced UTI events with *E. coli* and/or *Klebsiella* *spp*. Of these, 18% (69/389) had a least one UTI event with an ESBL-producing strain within the first year post-transplant (Figure 1). The baseline characteristics of the cohort grouped by the ESBL status are detailed in Table 1. Transplant recipients in both groups were in median 56 years old. In both the non-ESBL and the ESBL group, female sex was more common (60% versus 51%, respectively) but there were no significant differences between the groups (*p* = 0.16). There was also no difference with respect to the underlying renal diseases, with a special focus on those that may pose patients at higher risk for UTI events. In this contemporary cohort, both groups were mostly (89% versus 87%, respectively) treated with a maintenance immunosuppression consisting of tacrolimus, mycophenolate and steroids and 29% in both groups received an induction therapy with a T cell-depleting agent.

**Table 1:** Baseline characteristics of patients with *Escherichia coli* and/or *Klebsiella* *spp*. UTI in the first year post-transplant grouped according to ESBL status.

Parameter	No ESBL (*n* = 320)	ESBL (*n* = 69)	*p*-value
Recipient age	56 (47–63)	56 (46–65)	0.81
Female sex	192 (60%)	35 (51%)	0.16
Recipient renal disease ADPKD Diabetic Nephropathy Reflux/Pyelonephritis Other	75 (23%) 22 (7%) 29 (9%) 194 (61%)	21 (30%) 6 (9%) 5 (7%) 37 (54%)	0.56
RRT prior to transplantation HD PD None	228 (71%) 37 (12%) 54 (17%)	54 (78%) 5 (7%) 10 (15%)	0.46
Donor age	54 (43–63)	55 (47–64)	0.61
Deceased donor	200 (63%)	47 (68%)	0.38
Cold ischemia time [h]	7.2 (1.8–10.4)	8.6 (2.0–11.9)	0.12
CMV constellation High risk Intermediate risk Low risk Unknown	54 (17%) 204 (64%) 58 (18%) 4 (1%)	13 (19%) 44 (64%) 12 (17%) 0	0.80
Pre-transplant HLA-DSA	71 (22%)	12 (17%)	0.38
AB0 incompatible	22 (7%)	4 (6%)	0.75
A/B/DRB1 mismatches	4 (3–5)	4 (3–5)	0.43
A/B/DRB1-5/DQB1 mismatches (*n* = 355)	5 (4–7)	5 (4–7)	0.30
Induction therapy ATG/Thymoglobulin Basiliximab None	94 (29%) 223 (70%) 3 (1%)	20 (29%) 48 (70%) 1 (1%)	0.93
Maintenance immunosuppression FK/MPA/Pred CyA/MPA/Pred Other	286 (89%) 27 (9%) 7 (2%)	60 (87%) 7 (10%) 2 (3%)	0.84

ADPKD, autosomal polycystic kidney disease; RRT, renal replacement therapy; HD, Hemodialysis; PD, Peritoneal dialysis; CMV, cytomegalovirus; HLA-DSA, donor-specific HLA antibodies; ATG, anti-T cell globulin; Tac, tacrolimus; MPA, mycophenolic acid; Pred, prednisone; CyA, cyclosporine.

### 3.2 Major one-year outcomes according to ESBL status

**Figure 2:**
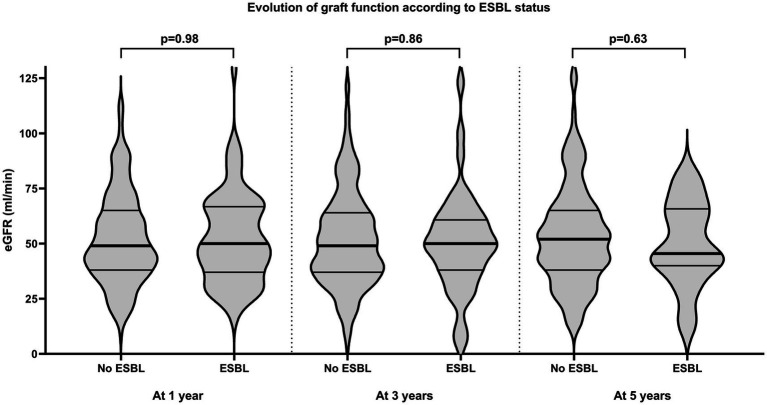
Evolution of graft function in patients with *Escherichia coli* and/*or Klebsiella* *spp*. UTI in the first year post-transplant grouped according to ESBL status, shown by violin plots (the lines represent the median (bold) as well as the interquartile ranges). eGFR, estimated glomerular filtration rate; ESBL, extended-spectrum beta-lactamase.

We compared major one-year outcomes among transplant recipients according to their grouped ESBL status (Table 2). Overall, graft loss and patients’ death were rare events in both groups and no statistically significant differences were observed (2.2% versus 2.9%, *p* = 0.72). Graft function at one year did not differ among the two groups (Table 2, Figure 2). Furthermore, we did not observe differences with respect to the occurrence of rejection. However, transplant recipients with at least one first-year UTI event with an ESBL-producing strain experienced more frequently colonization episodes (*p* = 0.0004). In addition, there was a significantly higher proportion of recurrent UTI in the ESBL group (18.4% versus 33.3%, respectively). Notably, the number of severe UTI, namely urosepsis episodes, was not statistically significant different among the two groups (*p* = 0.83).

**Table 2:** First-year outcomes in patients with *Escherichia coli* and/*or Klebsiella* *spp*. UTI in the first year post-transplant grouped according to ESBL status

Parameter	No ESBL (*n* = 320)	ESBL (*n* = 69)	*p*-value
Graft loss or death	7 (2.2%)	2 (2.9%)	0.72
Death	5 (1.6%)	1 (1.5%)	0.95
Graft loss	2 (0.6%)	1 (1.5%)	0.48
eGFR [ml/min]	49 (38–65)	50 (37–67)	0.98
Number of transplant biopsies None One Two More than two	137 (42.8%) 100 (31.3%) 58 (18.1%) 25 (7.8%)	29 (42.0%) 21 (30.4%) 14 (20.3%) 5 (7.3%)	0.98
Number of rejections None One Two or more	275 (85.9%) 39 (12.2%) 6 (1.9%)	58 (84.1%) 7 (10.1%) 4 (5.8%)	0.16
Number of colonization episodes	1 (0–2)	2 (1–3)	**0.0004**
Number of UTI None One Two More than two	86 (26.9%) 117 (36.6%) 58 (18.1%) 59 (18.4%)	19 (27.6%) 15 (21.7%) 12 (17.4%) 23 (33.3%)	**0.02**
UTI phenotype Only colonization Occasional UTI (1–2 UTI) Recurrent UTI (≥3 UTI)	86 (26.9%) 175 (54.7%) 59 (18.4%)	19 (27.6%) 27 (39.1%) 23 (33.3%)	**0.013**
Any Urosepsis episodes None One Two More than two	282 (88.2%) 32 (10%) 3 (0.9%) 3 (0.9%)	58 (84.1%) 9 (13.1%) 1 (1.4%) 1 (1.4%)	0.83

eGFR, estimated glomerular filtration rate; UTI, urinary tract infection.

*P*-values indicating significant results are shown in bold.

Within the ESBL group, infections with an ESBL-producing strain were in 34 (34/69; 49%) transplant recipients the first recorded UTI event. Among the other 35 patients, a majority (26/35; 74%) had previously received an antibiotic therapy for an antecedent non-ESBL UTI event. These non-ESBL UTI events were mostly (21/26; 81%) previous UTI or urosepsis episodes and in only 5 patients (5/26; 19%) previously treated colonization (data not shown).

### 3.3 Impact of ESBL-UTI on longer-term patient and graft survival and evolution of graft function

**Figure 3:**
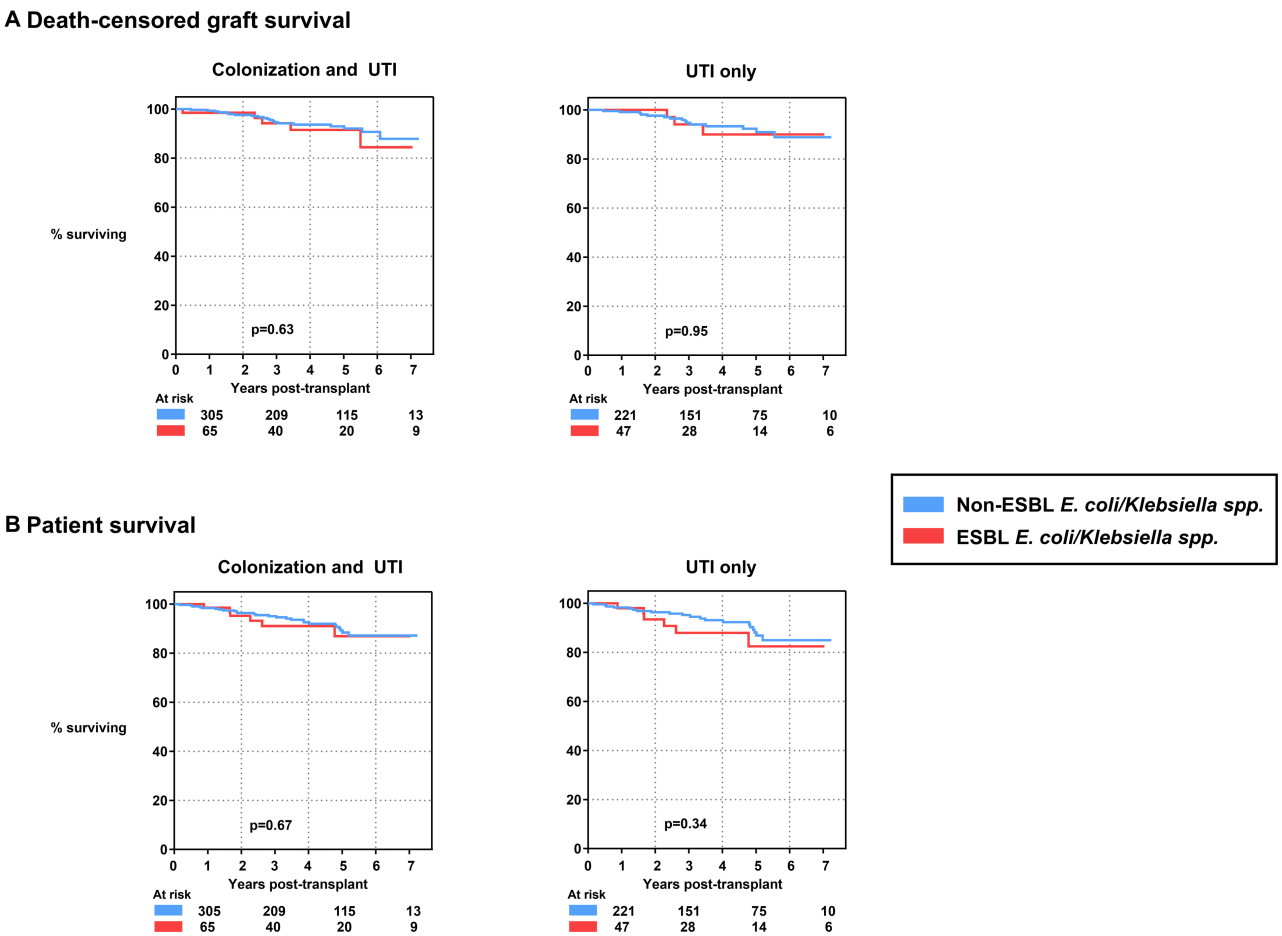
**(A)** Death-censored graft survival of kidney transplants experiencing first-year UTI events with and without ESBL-producing *E. coli* and/or *Klebsiella* *spp*., shown for colonization and UTI events (left) as well as UTI only (right). **(B)** Patient survival of kidney transplants experiencing first-year UTI events with and without ESBL-producing *E. coli* and/or *Klebsiella* *spp*., shown for colonization and UTI events (left) as well as UTI only (right). ESBL, extended-spectrum beta-lactamase; *E. coli, Escherichia coli*; UTI, urinary tract infection.

Then, we focused on the impact of UTI with ESBL-producing strains on the longer-term patient and allograft survival (Figure 3). Patients were followed for a median of 4.0 years (2.1–5.1 years). Both death-censored allograft survival and patient survival were not different among the two groups (*p* = 0.63 and *p* = 0.67, respectively). This finding did not change when we excluded patients who had only colonization episodes (n = 84; 28% and n = 18; 28%, respectively) but no UTI. Notably, the outcome of both groups was similar when compared to transplant recipients without any first-year UTI event (Supplementary Figure 1).

Beside patient and death-censored graft survival, we also investigated the evolution of graft function on the longer term. As expected, there was no statistically significant difference between the two groups at three and five years post-transplant (Figure 2).

### 3.4 Incidence of infections with *Escherichia coli* and/or *Klebsiella* *spp*. on the UTI level

In a next step, we focused on details of the infections on the UTI level. In the cohort consisting of 389 kidney transplants, 1,133 UTI events occurred in total. Of these, 825/1133 (73%) were caused by *E. coli* and/or *Klebsiella* *spp*. (Figure 1).

**Figure 4:**
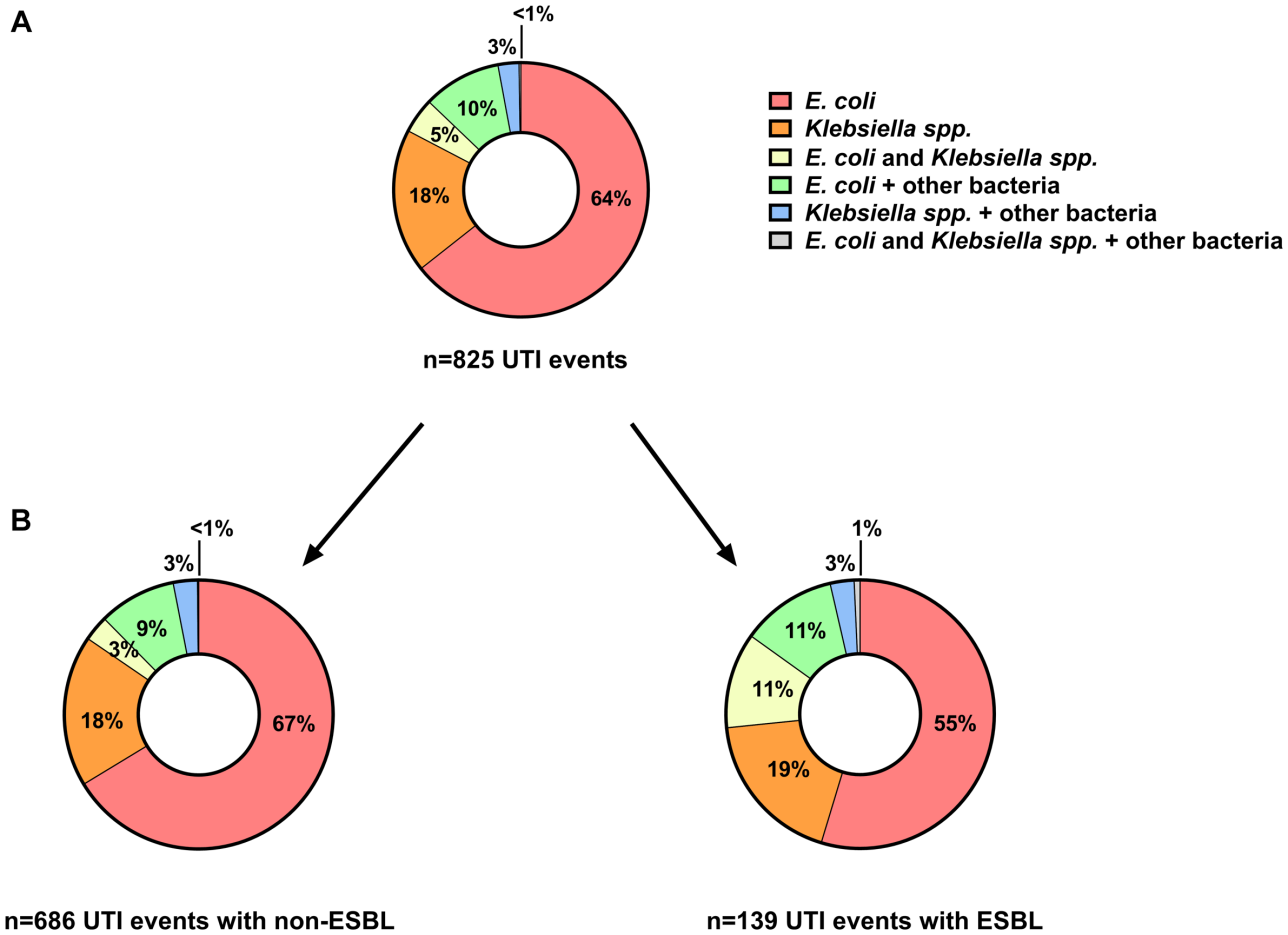
Distribution of *E. coli* and/or *Klebsiella* *spp*. in **(A)** all UTI events (n = 825) as well as separately shown in **(B)** UTI events without (n = 686) and with (n = 139) ESBL-producing strains. ESBL, extended-spectrum beta-lactamase; *E. coli, Escherichia coli*; UTI, urinary tract infection.

Most of these UTI events could be exclusively attributed to *E. coli* and/or *Klebsiella* *spp*. (719/825; 87%, Figure 4A). In about 5% of cases, both pathogens were found at the same time. If concomitant bacteria were present, these were mostly *Enterococcus* *spp*. (67/106; 63%, data not shown). As expected, the frequency of UTI events with *E. coli* was higher than with *Klebsiella* *spp*. (Figure 4A).

Regarding the clinical phenotypes observed among all UTI events caused by *E. coli* and/or *Klebsiella* *spp*. (non-ESBL and ESBL), 37% (307/825) were colonization and 56% (459/825) UTI episodes (Figure 1). Urosepsis episodes were considerably less frequent (7%, 59/825;).

**Figure 5:**
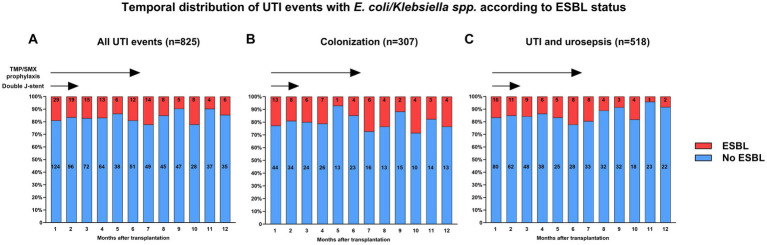
Temporal distribution of UTI events with *E. coli/Klebsiella* *spp*. grouped by the presence and absence of an ESBL-producing strain for **(A)** all UTI events as well as separately shown for **(B)** colonization and **(C)** UTI and urosepsis episodes. ESBL, extended-spectrum beta-lactamase; *E. coli, Escherichia coli*; TMP/SMP, trimethoprim-sulfamethoxazole; UTI, urinary tract infection.

Overall, ESBL-producing strains were detected in 139/825 (17%) of all UTI events caused by *E. coli* and/or *Klebsiella* *spp*. (Figure 4B). The distribution of *E. coli* and/or *Klebsiella* *spp*. among UTI events as compared with non-ESBL-producing strains was very similar (Figure 4B). We did not observe a difference in the proportion of UTI with ESBL-producing strains with respect to the clinical phenotype (62/307; 20% of colonization, 67/459; 15% of UTI, 10/59; 17% of urosepsis episodes, respectively, *p* = 0.13). In addition, the proportion of UTI events with an ESBL-producing strain remained rather stable over time (Figure 5).

### 3.5 Frequency of treatment and risk for hospitalization

Compared with UTI and urosepsis episodes that were almost always treated with antibiotics (100% in urosepsis and 99.4% in UTI), colonization was only treated in 66/307 episodes (21%). Moreover, when comparing colonization episodes with and without ESBL-producing strains, treatment frequency was very similar (53/245; 21.6% without and 13/62; 21.0% with ESBL, *p* = 0.91).

Within the dataset, information on the need for infection-related hospitalization was available in 97% of UTI events. Hospitalization was required in 97% of urosepsis and 28% of UTI events. In cases of colonization, hospitalization only rarely occurred (<3%). While urosepsis almost always prompted hospitalization regardless of the ESBL status (100 and 95.9%, respectively), there was a significantly higher proportion of hospitalizations in UTI with ESBL-producing strains (39% versus 26%, *p* = 0.04).

## 4 Discussion

In this nationwide multicenter study, we investigated the impact of UTI due to ESBL-producing *E. coli* and/or *Klebsiella* *spp*. on renal transplant outcomes. The key observation of this study is that occurrence of UTI with ESBL-producing strains did not affect allograft and patient survival. Furthermore, there was no difference in allograft function between patients with and without at least one UTI event with an ESBL-producing strain.

The results of this study suggest a limited clinical and predominantly epidemiological significance of ESBL-producing *Enterobacterales*. In this regard, our results are in part contradictory compared with results of previous studies pointing toward a lower patient survival, a higher case fatality rate as well as a higher virulence of infections caused by such resistant bacterial strains (13, 16, 17). Some reasons might explain these discrepancies. First, we hypothesize that the clinical overall awareness for ESBL-producing bacteria increased over the last 5−10 years, which might have influenced management of patients in case of a lacking clinical response following initial empiric therapy. Secondly, antibiotic resistance profiles are nowadays usually available within 24 to maximum 72 h, facilitating a timely adaptation of antibiotics. Third, the percentage of severe UTI events, namely urosepsis episodes, caused by ESBL-producing strains was rather low (in total 10 events) in our cohort. Therefore, we cannot exclude that a delay of appropriate treatment is more detrimental in this particular subgroup. However, the overall rather low frequency of urosepsis episodes of 7% in our cohort generally suggests that current post-transplant surveillance and instruction of patients is often able to prevent development of such severe infections (i.e., urosepsis), which might mitigate a potential risk conferred by ESBL production in this regard.

Consistent with the study of Bodro et al. we found that transplant recipients in the ESBL group had a higher proportion of recurrent UTI (12). In addition, there was a significantly higher proportion of hospitalizations required in UTI with ESBL-producing strains. These results underline the economic burden of disease (10, 18). It seems likely that, given the limited availability of outpatient parenteral antibiotic application services, intravenous application was often the main reason for hospitalization. Since this might have provoked a tendency toward a shorter treatment, it could also explain more recurrent UTI in the ESBL group. Facing lacking options for oral treatment in infections with ESBL-producing bacteria in many cases, improvement of management options by expansion of outpatient services for intravenous antibiotic administration as well as better antibiotic counseling in terms of optimal treatment duration are needed.

In this study, we observed that 18% of transplant recipients developing UTI with *E. coli* and/or *Klebsiella* *spp*. within the first year post-transplant experience at least one infection with an ESBL-producing strain. With respect to the whole cohort consisting of 1,482 transplants, this corresponds to an absolute frequency of 5%. The detected frequency is consistent with results of other studies reporting an overall prevalence of about 5% among kidney transplant recipients and a proportion of 20% among *Enterobacterales* in Europe (11, 19). Neither classical risk factors for UTI, such as female sex and the type of underlying renal disease (e.g., ADPKD, Diabetes, reflux nephropathy), nor the intensity of immunosuppression, such as an induction therapy with a T cell-depleting agent, were associated with the occurrence of UTI with an ESBL-producing strain. In addition, there was a rather stable monthly proportion of 10 to 20% of ESBL-UTI over time, making an influence of the antibiotic prophylaxis with trimethoprim-sulfamethoxazole for pneumocystis prevention as well as the DJ catheter *in situ* rather unlikely. Notably, we observed that the infection with an ESBL-producing strain was the first recorded UTI event in about 50% of transplant recipients ultimately developing such an UTI, suggesting that pre-existing unrecognized colonization or acquisition in the community might be underestimated risk factors. While pre-existing colonization could potentially be counterbalanced by the use of perioperative prophylaxis with carbapenems, future studies should focus on further delineating the mechanism of infection as well as the prevention of selection pressure conferred by antecedent antibiotic therapies (20, 21). In this regard, it is important to further study the wide range of virulence factors, especially since it was recently nicely shown that there is no regular pattern for ESBL production with respect to for instance type 3 fimbriae comprising an important superficial virulence factor (22, 23).

One particular strength of our study lies in the detailed analysis of a large and unselected multicenter cohort with a follow-up of a median of 4 years. Notably, our cohort is one of the largest focusing on UTI caused by ESBL-producing *Enterobacterales* in kidney transplant recipients. Furthermore, all patients studied were treated with contemporary immunosuppression. Another strength is the strict separation of the different UTI phenotypes (i.e., colonization, UTI and urosepsis) in our cohort.

However, our study is also subject to limitations. First, we had no information on the type and the lengths of antibiotics used for treatment of UTI. Therefore, treatment failure, especially in UTI with ESBL-producing *E. coli* and/or *Klebsiella* *spp*., cannot be ruled out and might have influenced the results with respect to the frequency of infections. Second, our analysis focuses only on UTI events occurring within the first year post-transplant. This decision was made based on the completeness of data as well as the fact that the first year is the period with the highest incidence of infections in general. Third, we focused only on UTI and can therefore not exclude that patients developed infections of other organs or colonization with ESBL-producing strains at other locations. However, an impact of other severe infections seems unlikely in light of the similar patient survival of both groups. Fourth, we had no information that allowed us to distinguish between upper and lower urinary tract infections. Additionally, we could not analyze complications of UTI such as the abscess development or obstruction. Lastly, the results of this study might have been influenced by local epidemiological factors and clinical practice, limiting its general validity in other countries.

In conclusion, overall 5% of all patients and about 20% of patients with UTI caused by *E. coli* and/or *Klebsiella* *spp*. develop at least one first-year UTI with an ESBL-producing strain. These infections are associated with a higher need for hospitalization but do not impact allograft function as well as allograft and patient survival.

## Data availability statement

The raw data supporting the conclusions of this article will be made available by the authors, without undue reservation.

## Ethics statement

The studies involving humans were approved by Ethics Committee of Northwestern and Central Switzerland (www.eknz.ch; project ID 2021-00360). The studies were conducted in accordance with the local legislation and institutional requirements. The participants provided their written informed consent to participate in this study.

## Author contributions

JB: Formal analysis, Investigation, Writing – original draft. MD: Writing – review & editing. DS: Writing – review & editing. LW: Writing – review & editing. DG: Writing – review & editing. OM: Writing – review & editing. FH: Writing – review & editing. DN: Writing – review & editing. AS: Writing – review & editing. KB: Writing – review & editing. TM: Writing – review & editing. TS: Writing – review & editing. NK: Writing – review & editing. SS: Conceptualization, Writing – review & editing. CW: Conceptualization, Formal analysis, Investigation, Writing – original draft.

## Group member of the Swiss Transplant Cohort Study

Patrizia Amico, John-David Aubert, Adrian Bachofner, Vanessa Banz, Sonja Beckmann, Guido Beldi, Christoph Berger, Ekaterine Berishvili, Annalisa Berzigotti, Pierre-Yves Bochud, Sanda Branca, Heiner Bucher, Anne Cairoli, Emmanuelle Catana, Yves Chalandon, Sabina De Geest, Sophie De Seigneux, Michael Dickenmann, Joëlle Lynn Dreifuss, Michel Duchosal, Thomas Fehr, Sylvie Ferrari-Lacraz, Jaromil Frossard, Christian Garzoni, Déla Golshayan, Nicolas Goossens, Fadi Haidar, Jörg Halter, Dominik Heim, Christoph Hess, Sven Hillinger, Hans Hirsch, Patricia Hirt, Linard Hoessly, Günther Hofbauer, Uyen Huynh-Do, Franz Immer, Michael Koller, Andreas Kremer, Christian Kuhn, Bettina Laesser, Frédéric Lamoth, Roger Lehmann, Alexander Leichtle, Oriol Manuel, Hans-Peter Marti, Michele Martinelli, Valérie McLin, Katell Mellac, Aurélia Merçay, Karin Mettler, Nicolas Müller, Ulrike Müller-Arndt, Beat Müllhaupt, Mirjam Nägeli, Graziano Oldani, Manuel Pascual, Jakob Passweg, Rosemarie Pazeller, Klara Posfay-Barbe, David Reineke, Juliane Rick, Anne Rosselet, Simona Rossi, Rössler, Silvia Rothlin, Frank Ruschitzka, Thomas Schachtner, Stefan Schaub, Alexandra Scherrer, Dominik Schneidawind, Aurelia Schnyder, Macé Schuurmans, Simon Schwab, Thierry Sengstag, Federico Simonetta, Jürg Steiger, Guido Stirniman, Ueli Stürzinger, Christian Van Delden, Jean-Pierre Venetz, Jean Villard, Julien Vionnet, Madeleine Wick, Markus Wilhlem, Patrick Yerly.
