# Supplementary figures and images for: Frequency and impact on renal transplant outcomes of urinary tract infections due to extended-spectrum beta-lactamase-producing Escherichia coli and Klebsiella species
Source: Front Med (Lausanne). 2024 Feb 15;11:1329778. doi: 10.3389/fmed.2024.1329778 (PMC10902035; doi:10.3389/fmed.2024.1329778)

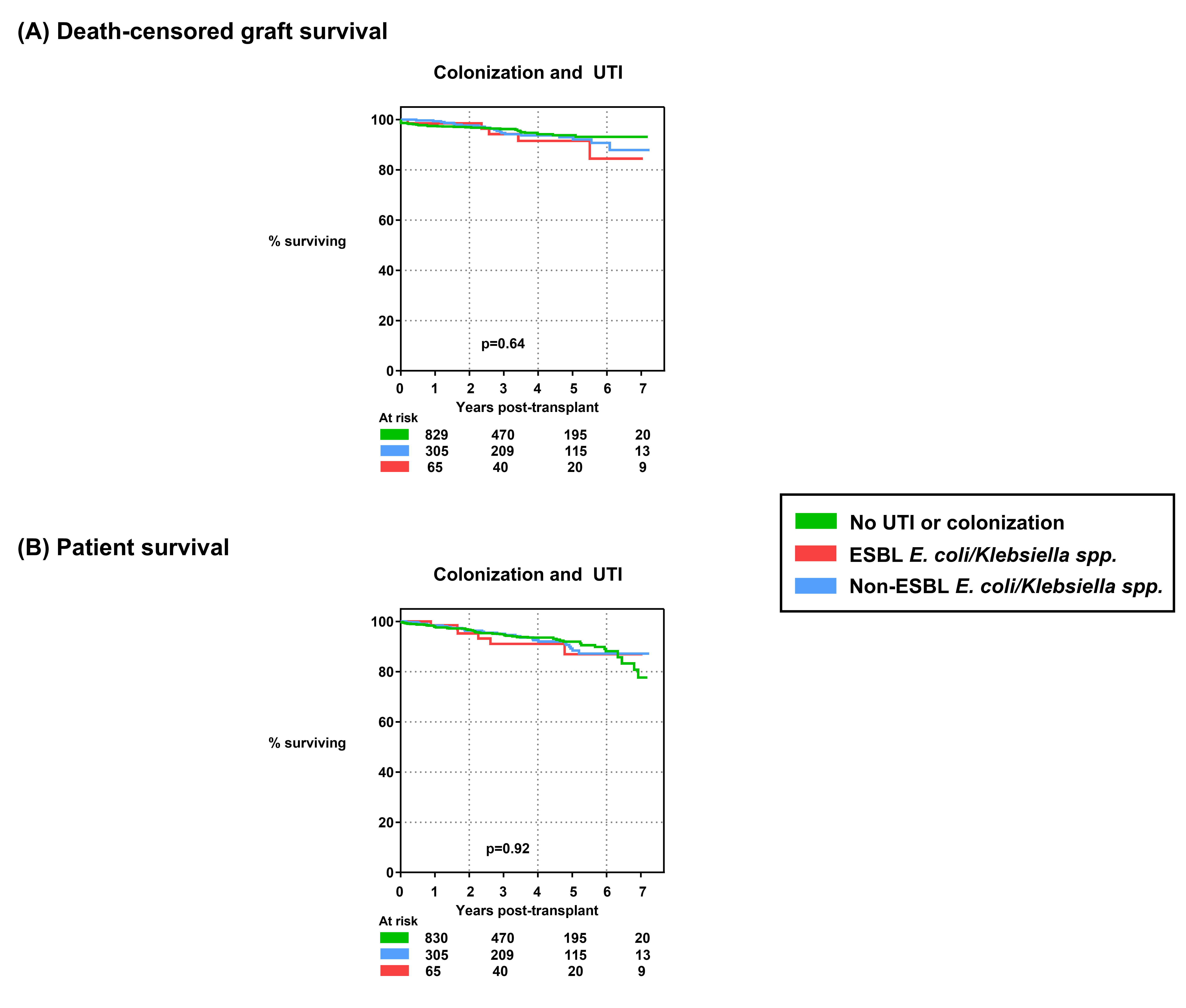

Supplement: Supplementary file 1 [file Image_1.TIF]
